# An isolated subcutaneous paraspinal pseudo-tumor—more to it than meets the eye—a case report and review of literature

**DOI:** 10.1093/jscr/rjad544

**Published:** 2023-10-21

**Authors:** Swapnil Keny, Nihar Modi, Aryan Keny, Nikhil Gokhale, Saurabh Yadav, Kalpana Azad

**Affiliations:** KB Bhabha Municipal Hospital, Bandra, Mumbai, Maharashtra, India; Department of Orthopaedics, KB Bhabha Municipal Hospital, Bandra, Mumbai, Maharashtra, India; Seth GS Medical College and KEM Hospital, Mumbai, India; Department of Orthopaedics, KB Bhabha Municipal Hospital, Bandra, Mumbai, Maharashtra, India; Department of Orthopaedics, KB Bhabha Municipal Hospital, Bandra, Mumbai, Maharashtra, India; Department of Pathology, KB Bhabha Municipal Hospital, Bandra, Mumbai, Maharashtra, India

**Keywords:** neurocysticercosis, paraspinal pseudo-tumor, Taenia Solium, subcutaneous cysticercosis, pediatric child

## Abstract

A 4-year-old male child presented to us with a paraspinal pseudo-tumor over the mid-back region with pain being his only symptom. On initial ultrasonography, it was presumed to be a nerve sheath tumor, but on an excision biopsy and histopathology, it proved to be a subcutaneous cysticercosis. Furthermore, an MRI of the brain showed a ring enhancing lesion with vasogenic edema, which confirmed the diagnosis of a neurologically symptomless neurocysticercosis. We treated the patient with albendazole and a short course of dexamethasone. There was complete resolution of the painful subcutaneous swelling, and the patient remained neurologically symptomless at all subsequent follow-ups. Resolution of the brain lesions was seen in the 6-month MRI follow-up. Although rare, orthopedic surgeons should consider the possibility of parasitic infections when dealing with small near-asymptomatic soft tissue paraspinal swellings of uncertain etiology. A thorough investigation in such cases can be lifesaving.

## Introduction

Benign spinal and paraspinal soft tissue disorders are very common. They can range from herniated or sequestrated disks, synovial cysts, and degenerative arthritis to rare infectious conditions like cysticercosis [1].

In developing countries like India, cysticercosis has become a major health concern. It is a parasite-borne disease caused by cysticercus cellulosae, which is the larval form of pork tapeworm, Taenia Solium [2, 3]. It predominantly infects the central nervous system, followed by the eyes, subcutaneous tissue, skeletal muscle, lungs, and heart [3, 4]. Because of the vague and nonspecific presentation, it poses a diagnostic difficulty to the treating doctor.

We present the case of a 4-year-old male child with dorsal paraspinal swelling, which initially seemed to be a case of an isolated subcutaneous cysticercosis, with no apparent neurological symptoms.

## Case report

**Figure 1:**
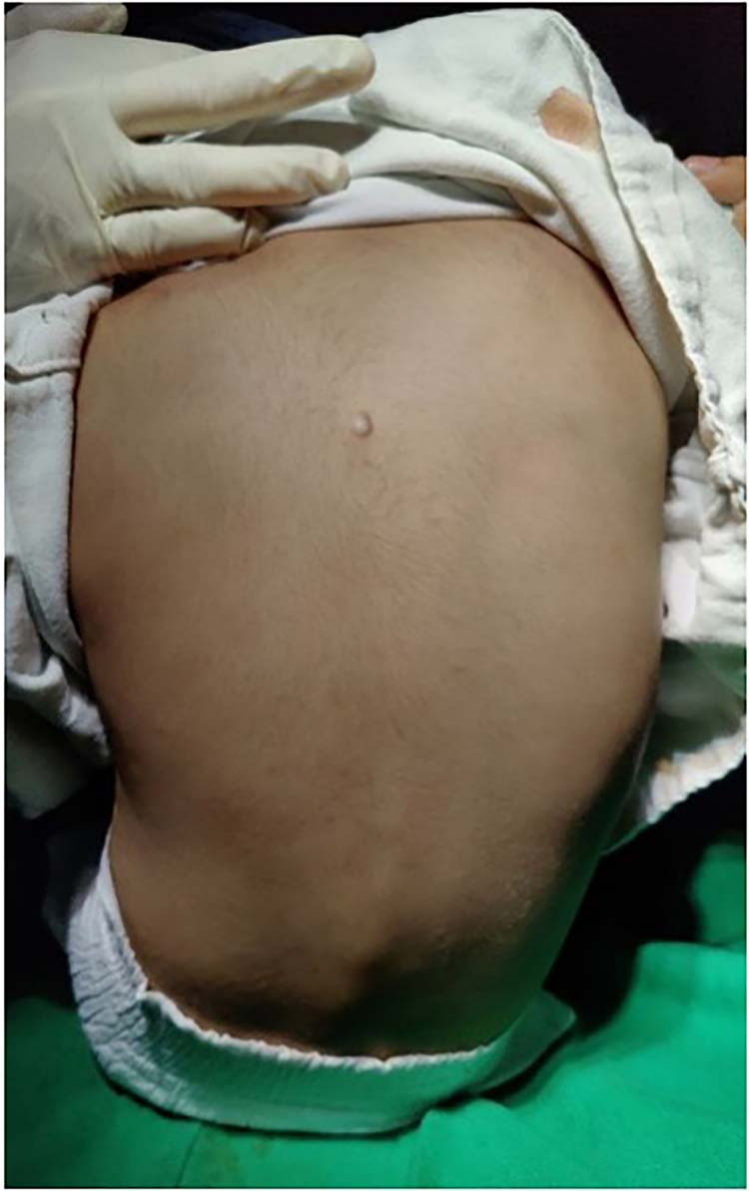
Pea-sized swelling in the mid-upper back region.

A 4-year-old male child, brought by his parents, presented to the orthopedic outpatient department of our hospital with swelling over the mid-upper back region (Fig. 1). The swelling was noticed initially by his parents at 6 months of age. It was initially painless, but the child developed a dull aching pain over the swelling 2 months before he presented to us. The swelling was initially very small, not visible to the naked eye, and gradually increased to the present size. The child had no history of trauma. There was no history of fever, weight loss, history of tuberculosis, or similar disease in the family.

On examination, we appreciated a pea-sized rounded mass with normal overlying skin. The swelling had no local rise of temperature and was non-tender, non-fluctuant, and non-reducible. The swelling was well-defined, firm, and in the subcutaneous plane. Clinically, the dimensions were 2 × 1 cm over the left paraspinal region, at the level of upper thoracic vertebrae.

**Figure 2:**
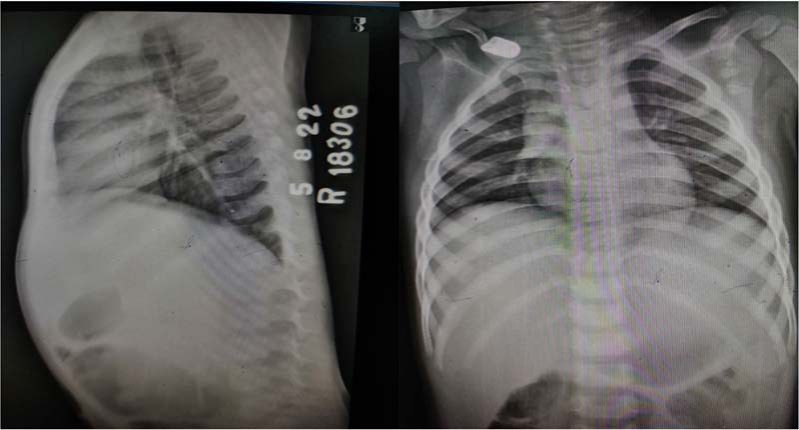
X-ray dorsal spine anteroposterior and lateral views, showing no significant abnormality.

**Figure 3:**
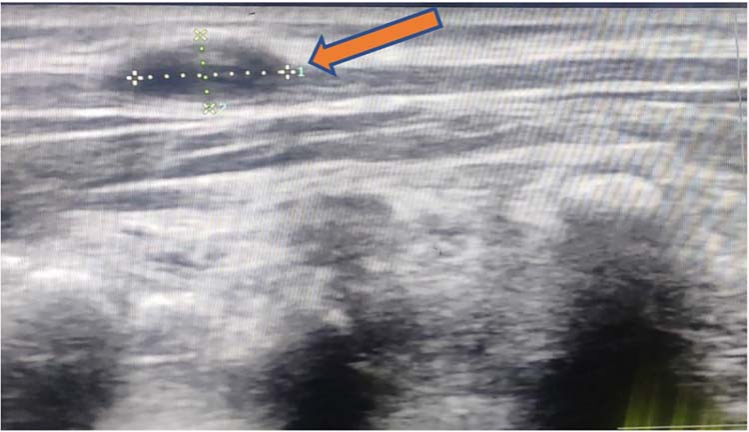
1.8 × 1.2 cm anechoic lesion in the subcutaneous plane with a small intramuscular extension.

X-ray chest and dorsal spine seemed within normal limits (Fig. 2). Ultrasonography (USG) of the mass revealed an anechoic 1.8 × 1.2 cm lesion with no internal vascularity or calcification (Fig. 3). It was found to be in the subcutaneous plane with a minor extension to the muscular plane over the mid-upper back region, giving it an impression of a nerve sheath tumor. The hemogram was within normal limits. At this stage, we had a differential diagnosis of a nerve sheath tumor in mind.

**Figure 4:**
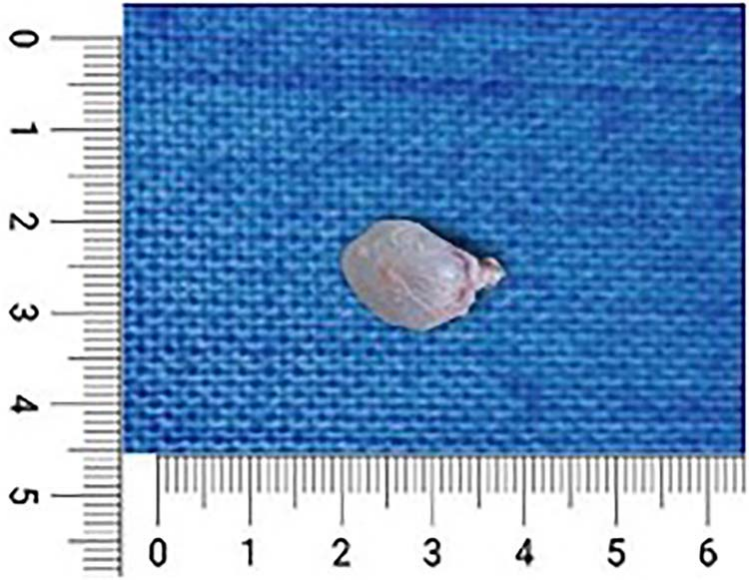
Intraoperative resected specimen during the open biopsy.

To confirm our diagnosis, we went ahead with an open biopsy. Under general anesthesia, with adequate aseptic precautions, a longitudinal incision of 4 × 1 cm was made over the swelling. The superficial fascia was dissected, and the swelling was found to be in the subcutaneous plane with a small component of paraspinal muscular infiltration. The mass along with its sac was removed, which was soft to firm with a jellylike consistency (Fig. 4). After a thorough wash, the incision was closed with Vicryl 2-0 and Monocryl 3-0. The incision healed well, and the postoperative period was insignificant. The sample obtained was sent for histopathological examination.

**Figure 5:**
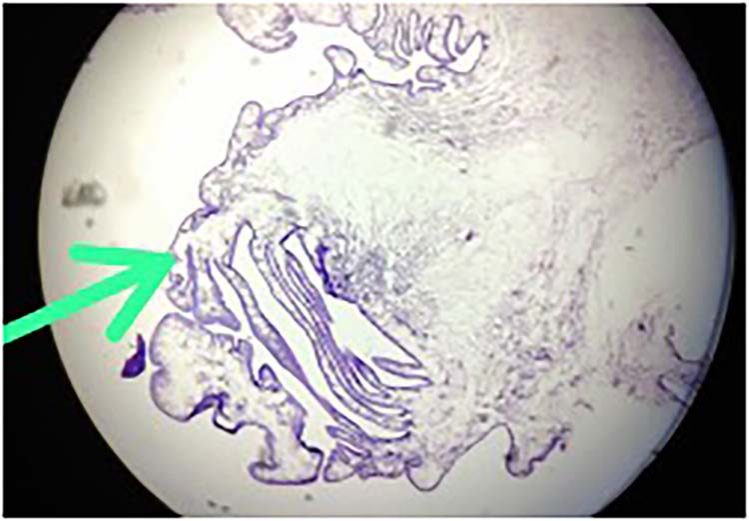
Hematoxylin-and-eosin-stained histopathology slide, showing cuticle, with subcuticular cells and chronic inflammatory infiltrate suggestive of cysticercosis.

**Figure 6:**
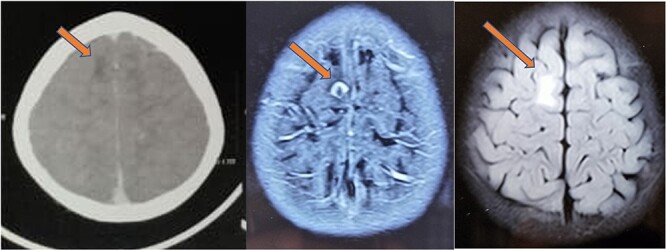
CT (left) showing a hypodense lesion in the right frontal lobe, T1 post-contrast MRI image (center) showing a ring enhancing lesion in the right frontal lobe and FLAIR image (right) showing non-enhancing vasogenic edema surrounding the lesion.

The histopathology report revealed the presence of a parasite composed of cuticle, subcuticular cells along with muscle, and chronic inflammatory infiltrate with a final impression of cysticercosis (Fig. 5). After this diagnosis, we reevaluated our history with more details. There was no history of headaches, seizures, or visual difficulties. The patient was found to be a vegetarian in diet. A pediatric physician evaluation was also done where a contrast-enhanced computed tomography (CECT) brain and ophthalmic examination were advised to evaluate for a possibility of neurocysticercosis. The ophthalmic examination was within normal limits, but the CECT showed a hypodense area in the right frontal lobe (Fig. 6). On further correlation with contrast MRI, a ring-enhancing lesion in the right paramedian frontal lobe surrounded by non-enhancing vasogenic edema was seen. Another small lesion was seen in the right external capsule surrounded by minimal edema (Fig. 6). Both lesions appeared to be granulomatous, giving an impression of neurocysticercosis. The child was treated with syrup albendazole 15 mg/kg per day in two divided doses for 1 month. A short course cover of dexamethasone 0.1 mg/kg per day started 1 day before albendazole was started, continued for a month, and then tapered over a week.

**Figure 7:**
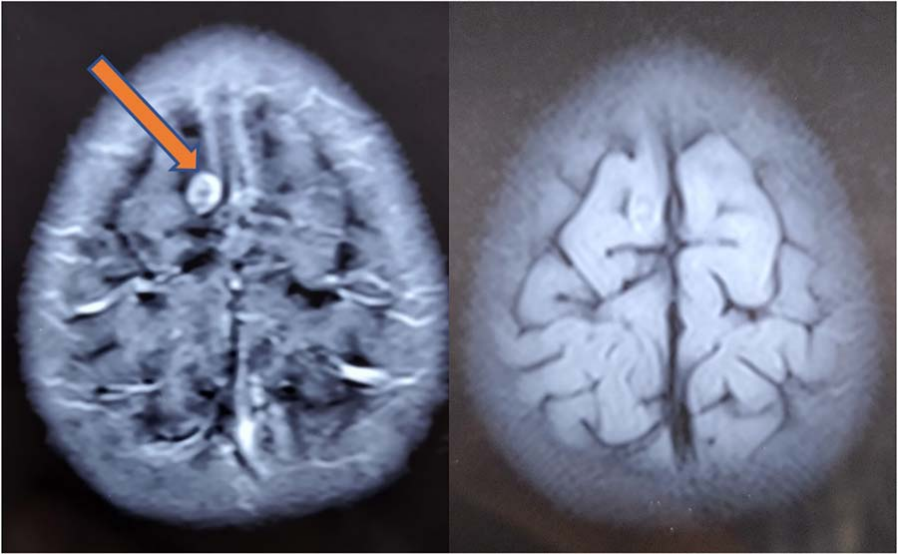
Repeat MRI at 6 months showing resolved changes with reduction in size of the lesion with resolution of perilesional edema.

At 1-month follow-up, the patient had complete resolution of pain and swelling. Local USG back showed no evidence of any remnant lesion. At the 6-month follow-up, an MRI of the brain was done that showed a major reduction in the size of the lesion and resolution of perilesional edema (Fig. 7). At 1-year follow-up, the patient was found to be completely symptom-free, managing his activities of daily life, very well.

## Discussion

Human cysticercosis is a parasitic infection caused by the larval stage of pork tapeworm, Taenia Solium [5]. It is endemic geographically to Southeast Asia, China, India, and Latin America, where unhygienic practices are rampant and pigs are raised as a source of food [6, 7].

Infection takes place by consumption of uncooked pork, vegetables, or water contaminated with human feces containing encysted larvae of Taenia Solium [4, 8]. Humans are definitive hosts, whereas pigs are the intermediate host. People of any age can be affected, but because there is a higher risk of fomite infection in children, they frequently suffer [4].

**Figure 8:**
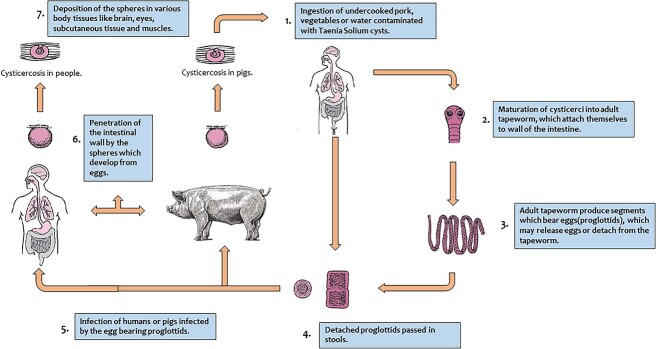
Life cycle of Taenia Solium.

After cysts are ingested by humans, gastric secretions in the human stomach break the outer wall of the cyst, and the head of the parasite (scolex), are released. The scolex anchors to the small intestinal mucosa and matures to form an adult tapeworm in 5–12 weeks. The tapeworm then sheds proglottids and eggs into the human faces, which remain undestroyed in the soil for days together, because of its thick covering shell. When food contaminated with this soil is ingested by humans, the breakdown of the eggshell by human stomach gastric secretions releases oncospheres (embryos). These oncospheres invade the intestinal wall, enter the mesenteric veins via the bloodstream and get lodged into various body tissues, and develop into cysticerci. Cysticerci can be found in any part of the body, but the brain, eyes, subcutaneous tissue, and muscles are where they most frequently appear [4, 8, 9] (Fig. 8).

When skeletal muscles are involved, they present as palpable cysticerci in the subcutaneous tissue. It can cause myositis and present as a palpable swelling or can have varied presentations depending on the site [10, 11]. When the central nervous system is involved (neurocysticercosis), it presents mostly symptomatically with symptoms ranging from headache, seizures, and intracranial hypertension to focal neurological deficits and cognitive decline [12].

Most isolated muscular and subcutaneous cysticercosis is symptomless and goes undiagnosed throughout the life of the patient. In a few rare instances, there can be a trauma to the cyst or the parasite inside it may die, releasing antigens that set off an immune reaction and inflammation, causing the cyst to become symptomatic [13]. Interestingly, Rangdal *et al*. [13], in their study, reported a similar case where a 45-year-old man developed pain and swelling over his calf after a traumatic injury, which was later proven to be a case of an intramuscular cysticercosis.

Clinical judgment of cysticercosis is very tough unless it is kept in the list of differential diagnoses. Pseudotumors causing a similar presentation include neurofibromas, lipomas, tuberculous lymphadenitis, pyomyositis, or epidermoid cysts [14, 15]. Despite a mid-back swelling, tuberculosis was kept very low in the list of differential diagnoses in our case, because of lack of constitutional symptoms and any tuberculous contact. Before the biopsy, a nerve sheath tumor was mainly on our differential diagnosis list, which proved to be incorrect after the biopsy.

Prior to the development of high-resolution sonography, USG was not frequently utilized in the diagnosis of muscle cysticercosis; however, it is now able to diagnose such disorders. The nonionizing and noninvasive nature of USG helps in establishing the possibility of such cases without the need for invasive techniques like a biopsy, which can then be managed conservatively with medications [4]. According to a recent research, the best assay for serological diagnosis of anticysticercal antibodies is the enzyme-linked immunoelectrotransfer blot. It was developed by the Centers for Disease Control and Prevention and has been shown to be 100% specific [16]. However, it was not considered in our case, as the disease was not suspected.

Medical therapy with praziquantel or albendazole is effective in reducing the size of the cystic lesions in neurocysticercosis [17]. The risk of neurological exacerbation during the 2nd–5th day of therapy because of death of the parasite and release of larval antigens has been documented. Hence, it is recommended to coadminister a steroid with albendazole or praziquantel to decrease inflammation [18]. The current guidelines of the American Academy of Neurology recommend the treatment of neurocysticercosis with albendazole and either prednisone or dexamethasone [17]. The management of our case was also on the same grounds as the above-mentioned literature. If not symptomatic, isolated subcutaneous and muscular cysticerci require no specific treatment [9]. The majority of authors agree that surgical excision is the optimal approach for symptomatic cysts outside the central nervous system [9, 19, 20].

Our study showed a similar result with a complete clinical and radiological resolution of the paraspinal lesion seen at the 1-month follow-up and thereafter.

To the best of our knowledge, our case is the only documented case report of neurocysticercosis presenting as an isolated subcutaneous swelling over the back with no neurological or ophthalmic manifestations seen typically in this condition, in a pediatric child.

## Conclusion

In an endemic zone like ours, neurocysticercosis may present as an asymptomatic isolated peripheral pseudotumor of uncertain etiology with absent neurological symptoms. High-resolution USG is a well-documented safe, cost-effective, and widely available method to document cysticercosis, and such cases should not be missed. Although rare, orthopedic surgeons should consider the possibility of parasitic infections when dealing with small soft tissue paraspinal swellings. This case report also shows how a thorough systemic investigation in such cases can be lifesaving.

## Author contributions

This letter describes individual contributions by the authors. Swapnil Keny (Supervision, Validation, Visualization), Nihar Modi (Writing original draft, Conceptualization, Methodology, Investigations), Aryan Keny (Writing the original draft, editing), Nikhil Gokhale (Investigations, Writing review and editing), Saurabh Yadav (Investigations, Review and editing), and Kalpana Azad (Review and editing)

## Conflict of interest statement

None declared.

## Funding

None declared.

## References

[1] Gross JM, Broski SM, Howe BM, Folpe AL. Paraspinal pseudoneoplasms: a series of 58 consultation cases emphasizing the importance of pathology-radiology correlation. Hum Pathol 2020;103:14–24. 32679051 10.1016/j.humpath.2020.07.012

[2] Kumar K, Indira V, Soni R, et al. Skeletal muscle cysticercosis. Int J Healthcare Biomed Res 2014;2:122–6.

[3] Thapa S, Lamichhane N, Joshi S. Isolated cysticercosis of sternocleidomastoid muscle: role of ultrasonography. Case Rep Infect Dis 2021;28:7102416. 10.1155/2021/7102416 PMC8492260 34621553

[4] Ramraje S, Bhatia V, Goel A. Solitary intramuscular cysticercosis-a report of two cases. Australas Med J 2011;4:58–60. 23393502 10.4066/AMJ.2011.483 PMC3562971

[5] Singh R, Kumar S, Galagali JR, Ramakrishnan N. Solitary cysticercosis of sternocleidomastoid muscle: a rare entity. Int J Otorhinolaryngol Head Neck Surg 2016;2:277–9.

[6] Lozano R, Naghavi M, Foreman K, et al. Global and regional mortality from 235 causes of death for 20 age groups in 1990 and 2010: a systematic analysis for the global burden of disease study 2010. Lancet 2012;380:2095–128. 23245604 10.1016/S0140-6736(12)61728-0 PMC10790329

[7] Sharma G, Ghode R. High resolution ultrasonography in isolated soft tissue and intramuscular cysticercosis. Int J Res Med Sci 2016;4:42–6.

[8] Naren Satya SM, Mayilvaganan KR, Amogh VN, Balakrishna BV, Gautam MS, Prathyusha IS. A classic case of subcutaneous cysticercosis: a rare case with sonological findings and review of literature. Pol J Radiol 2016;81:478–82. 27781073 10.12659/PJR.898408 PMC5056536

[9] Meena D, Gupta M, Jain VK, Arya RK. Isolated intramuscular cysticercosis: clinicopathological features, diagnosis and management - a review. J Clin Orthop Trauma 2016;7:243–9. 28053392 10.1016/j.jcot.2016.06.016 PMC5197059

[10] Sharma R, Gautam P, Kumar S, Elhence P, Bansal R, Gupta G. Isolated cysticercosis cellulosae of sternocleidomastoid muscle: a case report with review of literature. Indian J Otolaryngol Head Neck Surg 2011;63:127–30. 22754863 10.1007/s12070-011-0140-y PMC3146659

[11] Ramesh V . Cysticercosis. Int J Dermatol. 1984;23:348–50. 10.1111/j.1365-4362.1984.tb04067.x 6746185

[12] Garcia HH, Nash TE, Del Brutto OH. Clinical symptoms, diagnosis, and treatment of neurocysticercosis. Lancet Neurol 2014;13:1202–15. 25453460 10.1016/S1474-4422(14)70094-8 PMC6108081

[13] Rangdal SS, Prabhakar S, Dhatt SS, Prakash M, Dhillon MS, Dhillon. Isolated muscular cysticercosis: a rare pseudotumor and diagnostic challenge, can it be treated nonoperatively? A report of two cases and review of literature. J Postgrad Med Edu Res 2012;46:43–8.

[14] Jankharia BG, Chavhan GB, Krishnan P, Jankharia B. MRI and ultrasound in solitary muscular and soft tissue cysticercosis. Skeletal Radiol 2005;34:722–6. 16167132 10.1007/s00256-005-0954-3

[15] Despommier DD . Tapeworm infection — the long and the short of it. N Engl J Med 1992;327:727. 1495527 10.1056/NEJM199209033271011

[16] Hubert K, Andriantsimahavandy A, Michault A, Frosch M, Mühlschlegel FA. Serological diagnosis of human cysticercosis by use of recombinant antigens from Taenia Solium cysticerci. Clin Diagn Lab Immunol 1999;6:479–82. 10391846 10.1128/cdli.6.4.479-482.1999 PMC95711

[17] Rizvi SA, Saleh AM, Frimpong H, Al Mohiy HM, Ahmed J, Edwards RD, et al. neurocysticercosis: a case report and brief review. Asian Pac J Trop Med 2016;9:100–2. 26851797 10.1016/j.apjtm.2015.12.020

[18] Singhi P, Saini AG. Pediatric neurocysticercosis. Indian J Pediatr 2019;86:76–82. 28929415 10.1007/s12098-017-2460-8

[19] Abdelwahab IF, Klein MJ, Hermann G, Abdul-Quader M. Solitary cysticercosis of the biceps brachii in a vegetarian: a rare and unusual pseudotumor. Skeletal Radiol 2003;32:424–8. 12730733 10.1007/s00256-003-0638-9

[20] Ogilvie CM, Kasten P, Rovinsky D, Workman KL, Johnston JO. Cysticercosis of the triceps--an unusual pseudotumor: case report and review. Clin Orthop Relat Res 2001;382:217–21. 10.1097/00003086-200101000-00029 11153991

